# Editorial: Precision treatments for patients with obesity

**DOI:** 10.3389/fmed.2024.1423460

**Published:** 2024-05-29

**Authors:** Héctor Isaac Rocha-González, Ashuin Kammar-García, Ariana Vargas-Castillo, Juan Gerardo Reyes-García

**Affiliations:** ^1^Sección de Estudios de Posgrado e Investigación, Escuela Superior de Medicina, Instituto Politécnico Nacional, Ciudad de México, Mexico; ^2^Dirección de Investigación, Instituto Nacional de Geriatría, Ciudad de México, Mexico; ^3^Dana-Faber Cancer Institute, Harvard Medical School, Boston, MA, United States

**Keywords:** interindividual variability, obesity, personalized medicine, precision treatments, risk factors

Obesity is regarded as a complex and highly prevalent condition of multifactorial origin, which is associated with an increase in the risk of multiple chronic and incapacitating diseases and a decrease in life expectancy (1). This conceptualization would seem to integrate the available information on the multiple and redundant regulatory mechanisms of body energy balance, the numerous genetic, environmental, psychological, socioeconomic and cultural factors that lead to obesity and the associated health risks, and the alarming statistics on the prevalence of this disease (2–4).

The extraordinary complexity of obesity is proportional to the lack of suitable guides for a rational and appropriate treatment plan to reach weight loss goals in the short and long term, and also to the deficiency in the knowledge about special cares aimed at the patient with obesity suffering from various diseases.

The Research Topic titled: “*Precision Treatments for Patients with Obesity*,” was conceived with the intention of including scientific contributions that offer valuable insights into the treatment of patients with obesity, who are subject to various relevant conditions, as well as on potential factors that influence the genesis or the efficacy of obesity therapy and the improvement of personalized management of patient with obesity.

The experience with the current options for treating obesity, whether behavioral modification of lifestyle, pharmacotherapy, and bariatric surgery has shown that the effectiveness and risk of these strategies increase in the same order, in contrast to their accessibility, and that in all of them, a high level of interindividual variability in the amount of weight loss is observed. Furthermore, despite the imperative of implementing personalized strategies for treating obesity, there are currently no prospective studies that demonstrate that an individualized approach based on genotype or phenotype will yield uniform efficacy success (5).

Interestingly, other authors have postulated that the impact of this complex interconnection of factors that limit effective weight loss can be evaluated and projected early by assessing the response to short-term pharmacological treatments, considering as combined predictive factors the weight reduction achieved at the first month and the eventual development of tolerance to the treatment (6, 7). Naturally, the study of the potential impact of these predictive factors on the new monotherapy or combined pharmacological modalities, and even behavioral modification regimens, could be used to generate the basis for a more rational and defined anti-obesity treatment strategies.

Other studies included in this topic emphasize the significance of examining individual factors in the identification of risk factors for obesity, or those whose incorporation could be beneficial for the successful management of obesity.

Some medications, including antidiabetic and antidepressant drugs, are well known to produce weight gain, including antidepressant drugs (8). Segura-Cervantes et al. contributed with a non-clinical study examining the metabolic and body composition consequences of antibiotic administration in newborn rats. The administration of antibiotics frequently used in neonatal intensive care units to rats at birth was associated with an increase in weight gain due to fat accumulation and a deficient regulation of carbohydrates and lipids. These effects were not homogeneous for the antibiotics tested, and they were considered to be likely related to changes in the microbiota. The authors emphasized the need to develop careful protocols for the selection of antibiotics to be used in newborns as a goal to reduce metabolic consequences and the risk of obesity and diabetes later in life.

Lifestyle interventions for weight loss based on diet and exercise are the cornerstone of obesity therapy (9). Caicedo-Trujillo et al. presented a review of inspiratory muscle training in patients with obesity. They found that this approach improves physical capacity and inspiratory muscle strength without significant changes in lung function, body mass index, or metabolic parameters. The use of this strategy could be useful in subjects with obesity and with impaired exercise tolerance and poor therapy adherence.

Sarzani et al. collaborated with a narrative review on the accumulation of visceral adipose tissue as the primary cause of numerous cardiometabolic consequences. The authors emphasize the significance of adopting an adipocentric approach to address early and intensive visceral adipose tissue excess, rather than awaiting the onset of potentially hazardous cardiovascular, cerebrovascular, and renal adiposity-related conditions. Furthermore, the authors suggested that the combination of the innovative drugs glucagon-like peptide-1 receptor agonists and sodium/glucose cotransporter-2 inhibitors would be an effective initial treatment for obesity.

The other study by Martínez-Camacho et al. focused on a narrative review to examine the physical and respiratory care required by a critically ill patient with obesity. This type of patients is subject to metabolic stress and the consequent alterations in resting energy expenditure, hyperglycemia, loss of muscle mass, and issues related to mental health and cognitive functions. In this study, the authors emphasize the importance of having a multidisciplinary team involved in the early rehabilitation program and providing adequate ventilatory support.

Perichart-Perera et al. presented a protocol aimed at evaluating a multicomponent nutritional intervention for the prevention of gestational diabetes and infant growth and neurodevelopment impairment, based on the rationale that obesity is associated with micronutrient deficiencies and that the internal environment of the pregnant woman with obesity is characterized by inflammation and oxidative stress and that intensive nutritional care, additional to routine care, could help in the prevention of gestational diabetes mellitus, other perinatal outcomes, maternal and newborn nutritional status, infant growth, adiposity, and neurodevelopment compared to usual care in pregnant women with obesity.

In conclusion, this topic research provides insight into the study of a number of variables that could influence the early development of obesity or associated comorbidities, but also those related to the establishment of specific strategies to improve adherence and the foundation of expectations of treatment effectiveness. This type of effort will undoubtedly encourage the advancement and development of studies that attempt to tailor treatments to the obese subpopulation, as an essential part of the growth and development of precision medicine.

## Author contributions

HR-G: Writing – original draft, Writing – review & editing. AK-G: Writing – review & editing. AV-C: Writing – review & editing. JR-G: Writing – original draft, Writing – review & editing.
